# Stimulation of Glia Reveals Modulation of Mammalian Spinal Motor Networks by Adenosine

**DOI:** 10.1371/journal.pone.0134488

**Published:** 2015-08-07

**Authors:** David Acton, Gareth B. Miles

**Affiliations:** School of Psychology and Neuroscience, University of St Andrews, Fife, United Kingdom; University of Edinburgh, UNITED KINGDOM

## Abstract

Despite considerable evidence that glia can release modulators to influence the excitability of neighbouring neurons, the importance of gliotransmission for the operation of neural networks and in shaping behaviour remains controversial. Here we characterise the contribution of glia to the modulation of the mammalian spinal central pattern generator for locomotion, the output of which is directly relatable to a defined behaviour. Glia were stimulated by specific activation of protease-activated receptor-1 (PAR1), an endogenous G-protein coupled receptor preferentially expressed by spinal glia during ongoing activity of the spinal central pattern generator for locomotion. Selective activation of PAR1 by the agonist TFLLR resulted in a reversible reduction in the frequency of locomotor-related bursting recorded from ventral roots of spinal cord preparations isolated from neonatal mice. In the presence of the gliotoxins methionine sulfoximine or fluoroacetate, TFLLR had no effect, confirming the specificity of PAR1 activation to glia. The modulation of burst frequency upon PAR1 activation was blocked by the non-selective adenosine-receptor antagonist theophylline and by the A_1_-receptor antagonist 8-cyclopentyl-1,3-dipropylxanthine, but not by the A_2A_-receptor antagonist SCH5826, indicating production of extracellular adenosine upon glial stimulation, followed by A_1_-receptor mediated inhibition of neuronal activity. Modulation of network output following glial stimulation was also blocked by the ectonucleotidase inhibitor ARL67156, indicating glial release of ATP and its subsequent degradation to adenosine rather than direct release of adenosine. Glial stimulation had no effect on rhythmic activity recorded following blockade of inhibitory transmission, suggesting that glial cell-derived adenosine acts via inhibitory circuit components to modulate locomotor-related output. Finally, the modulation of network output by endogenous adenosine was found to scale with the frequency of network activity, implying activity-dependent release of adenosine. Together, these data indicate that glia play an active role in the modulation of mammalian locomotor networks, providing negative feedback control that may stabilise network activity.

## Introduction

There is now considerable evidence from electrophysiological and Ca^2+^-imaging studies that glia can both respond to activity at the synapses they enwrap with elevations in cytosolic Ca^2+^ and modulate the excitability of neighbouring neurons via the Ca^2+^-dependent release of so-called gliotransmitters [1,2]. However, the importance of these activities for the operation of neural networks and in shaping behaviours remains controversial [3–6]. In this study we examine the role of gliotransmission in spinal motor networks. These networks coordinate the rhythmic activation of flexor and extensor muscles within and between limbs during locomotion, and for this reason their output is immediately relatable to a defined behaviour [7].

Like other central pattern generators (CPGs) controlling stereotyped motor behaviours, spinal motor networks are subject to extensive neuromodulation, allowing network output to be varied according to the requirements of different environmental conditions, physiological states and developmental stages [8,9]. Although many modulators are neuronal in origin, previous studies have reported modulation of spinal cord and brainstem CPGs following release of glutamate and the purines ATP and adenosine from glia [10–12].

Adenosine is among the most widespread neuromodulators in the nervous system and participates in diverse processes in health and disease [13]. Modulatory adenosine may be released from cells directly or else result from the hydrolysis of ATP by extracellular ectonucleotidases [14–16]. Several studies have detected Ca^2+^-dependent release of ATP from glia, with subsequent degradation of ATP to adenosine and activation of neuronal A_1_ or A_2A_ adenosine receptors [17–20].

Adenosinergic modulation has previously been detected in motor networks of the spinal cord and brainstem. In the spinal cord of *Xenopus* tadpoles, ATP released during episodes of swimming serves to excite the locomotor CPG and extend the duration of bouts of swimming [21,22]. As swimming progresses, ATP is hydrolysed to adenosine, which activates A_1_ receptors to drive down network activity [23]. Similarly, we have recently shown that adenosine derived from the hydrolysis of ATP acts on A_1_ receptors to reduce the frequency of locomotor-related bursting in the spinal locomotor CPG of postnatal mice [12]. Spinal locomotor networks of postnatal rats are also modulated by adenosine but may be less sensitive compared to equivalent networks in mice [24]. In mice, modulation of locomotor-related activity by endogenous adenosine is abolished following pharmacological ablation of glia, indicating that glia rather than neurons are the principal source of modulatory adenosine in murine spinal motor networks [12]. In support of these observations, excitatory transmission at synapses onto ventral horn interneurons is augmented following chelation of Ca^2+^ in neighbouring glia; conversely, it is depressed by a mechanism involving both ATP hydrolysis and activation of A_1_ receptors when neighbouring glia are stimulated [20]. Mammalian brainstem networks controlling respiration, another rhythmic motor behaviour, are also depressed under the influence of adenosine. As in the spinal cord of *Xenopus* tadpoles, inhibition of network activity by adenosine is reported to follow ATP-mediated excitation [25]. In addition, adenosine exerts a tonic depression of network activity that is most evident in foetal stages [26–29].

In the present study we investigate whether direct stimulation of glial cells leads to modulation of ongoing locomotor-related network activity in spinal cord preparations isolated from postnatal mice and whether any such modulation involves adenosinergic signalling. We demonstrate that applying a stimulus known to enhance glial Ca^2+^ signalling results in the modulation of network activity, and that this entails the secretion by glia of ATP and its subsequent hydrolysis to adenosine. Interestingly, we find no evidence of other glial cell-derived modulators affecting spinal locomotor networks. We also provide evidence that adenosinergic modulation of locomotor networks scales with network activity, likely reflecting proportional release of adenosine. This implies that glia possess a mechanism for detecting activity in adjacent neurons, a key element of the tripartite synapse model [30], and that they provide negative feedback to regulate the output of spinal motor circuitry. Together, our findings suggest that adenosine is the primary glial cell-derived modulator of spinal motor networks and implicate glia as active participants in the modulation of these networks and thus of locomotor behaviour.

## Methods

### Ethics Statement

All procedures performed on animals were conducted in accordance with the UK Animals (Scientific Procedures) Act 1986 and were approved by the University of St Andrews Animal Welfare and Ethics Committee.

### Tissue preparation

For physiological experiments, spinal cords were isolated from postnatal day (P)1-P4 C57BL/6 mice as previously described [31]. In summary, animals were killed by cervical dislocation, decapitated and eviscerated before being transferred to a dissection chamber containing artificial cerebrospinal fluid (aCSF; equilibrated with 95% oxygen, 5% carbon dioxide, ~4°C). Spinal cords were then isolated between midthoracic and upper sacral segments, and ventral and dorsal roots were trimmed.

### Ventral root recordings

Isolated spinal cords were pinned ventral-side up in a recording chamber perfused with aCSF (equilibrated with 95% oxygen, 5% carbon dioxide; RT) at 10 ml/min. Glass suction electrodes were attached to the first or second lumbar ventral roots (L_1_, L_2_) on each side of the spinal cord to record flexor-related activity. In some experiments a further suction electrode was attached to the fifth lumbar ventral root (L_5_) to record the corresponding extensor-related activity. Locomotor-related activity was evoked by bath application of *N*-methyl-D-aspartic acid (NMDA; 5 μM), 5-hydroxytryptamine (5-HT; 10 μM) and dopamine (DA; 50 μM), unless otherwise stated, and was characterised by rhythmic bursting alternating contralaterally between upper ventral roots and ipsilaterally between upper ventral roots and L_5_. For disinhibited preparations [12,32], strychnine (1 μM) and picrotoxin (60 μM) were applied to evoke rhythmic bursting that was synchronous in all roots. In some experiments theophylline (20 μM); SCH58261 (25 μM); 8-cyclopentyl-1,3-dipropylxanthine (DPCPX; 50 μM); ARL67156 (50 μM); methionine sulfoximine (MSO; 100 μM) and glutamine (1.5 mM); or fluoroacetate (FA; 5 mM) and glutamine (1.5 mM) were bath-applied upon the onset of locomotor-related bursting at concentrations previously employed in this preparation [12]. Any drugs present during the control period were also present during TFLLR application and washout. In all experiments, stable rhythmic bursting was established over a period of ~1 h prior to bath-application of the PAR1-specific agonist TFLLR [33,34]. Rhythmic bursting was considered stable when the frequency, amplitude and duration of bursts were unchanged over several minutes. TFLLR application was limited to 5 min, consistent with methods previously employed [33,34]. Data were amplified and filtered (band-pass filter 30–3,000 Hz, Qjin Design) and acquired at a sampling frequency of 6 kHz with a Digidata 1440A analog-digital converter and Axoscope software (Molecular Devices, Sunnyvale, CA). Custom-built amplifiers (Qjin Design) enabled simultaneous online rectification and integration (50-ms time constant) of raw signals.

### Data analysis

Data were analysed off-line with DataView software (courtesy of Dr W.J. Heitler, University of St Andrews). Ventral-root bursts were identified from rectified/integrated traces and their instantaneous frequencies, peak-to-peak amplitudes, and durations were then measured from the corresponding raw traces. Amplitude was measured as a non-calibrated unit and is presented here as an arbitrary unit (a.u.). For time-course plots, data were averaged across 1-min bins, or 2-min bins for disinhibited preparations, and normalised to a 10-min pre-control period to permit comparison between preparations. Duty cycle was calculated as burst duration divided by cycle period. Statistical comparisons were performed on raw data averaged over 3-min periods for standard preparations or 6-min periods for disinhibited preparations. Data were analysed with repeated-measures ANOVA, one-way ANOVA, or Student’s *t*-tests. Bonferroni post-hoc tests were applied to pairwise comparisons. Sphericity was assessed with Mauchly’s test as appropriate, and Greenhouse-Giesser corrections were applied where necessary. *p* values < 0.05 were considered significant. Cohen’s *d* effect size was also determined where appropriate [35]. Tests were performed in SPSS Statistics for Windows, Version 21.0 (IBM Corp. Armonk, NY) or Excel 2013 (Microsoft Corp. Redmond, WA).

### Immunohistochemistry

P4-P11 C57BL/6 mice were dissected as above, and spinal cords were incubated in fixative containing 4% (w/v) paraformaldehyde and 0.1 M phosphate buffer (pH 7.4) for 16 h at 4°C, before being washed with 0.1 M phosphate-buffered saline (PBS; pH 7.4) and stored in PBS at 4°C. Slices from segments L_1_-L_3_ were cut at a thickness of 50 μm on a vibratome (Leica, Nussloch, Germany). Free-floating slices were incubated for 48 h at 4°C in solution containing primary antibodies, PBS, 1% (w/v) bovine serum albumin (BSA) and 0.1% (v/v) Triton X-100. Slices were then washed with PBS and incubated for 24 h at 4°C in solution containing secondary antibodies, PBS and 1% BSA. Slices were washed again with PBS and mounted with Vectashield (Vector Labs, Peterborough, UK). The stained tissue was examined with an epifluorescence microscope and structured illumination (Imager.M2 fitted with ApoTome.2, Carl Zeiss Microscopy, Göttingen, Germany). Primary antibodies were used at the following concentrations: 1:100; chicken polyclonal anti-glial fibrillary acidic protein (GFAP; Aves GFAP) [36–38], 1:100; chicken polyclonal anti-microtubule-associated protein 2 (MAP2; Millipore AB5543) [39–41], 1:200; rabbit polyclonal anti-protease activated receptor-1 (PAR1; Bioss bs-0828R) [42]. Secondary antibodies were used at the following concentrations: polyclonal anti-chicken FITC (Aves F-1005), 1:200; polyclonal anti-rabbit Cy3 (Jackson Immunoresearch 111-165-003), 1:500.

### Drug and Solution Preparation

The aCSF used for dissections and recordings contained (in mM) 127 NaCl, 26 NaHCO_3_, 10 glucose, 3 KCl (unless otherwise stated), 2 CaCl, 1.25 NaH_2_PO_4_, and 1 MgCl_2_. TFLLR, theophylline, MSO, FA and glutamine were supplied by Sigma-Aldrich (Poole, UK); DPCPX and SCH58261 were supplied by Abcam (Cambridge, UK); ARL67156 was supplied by Tocris Bioscience (Bristol, UK). All drugs were dissolved in reverse-osmosis water, except picrotoxin, DPCPX and SCH58261, which were dissolved in DMSO. The concentration of DMSO in working solutions did not exceed 0.1% (v/v).

## Results

### Stimulation of glia modulates locomotor network output

**Fig 1:**
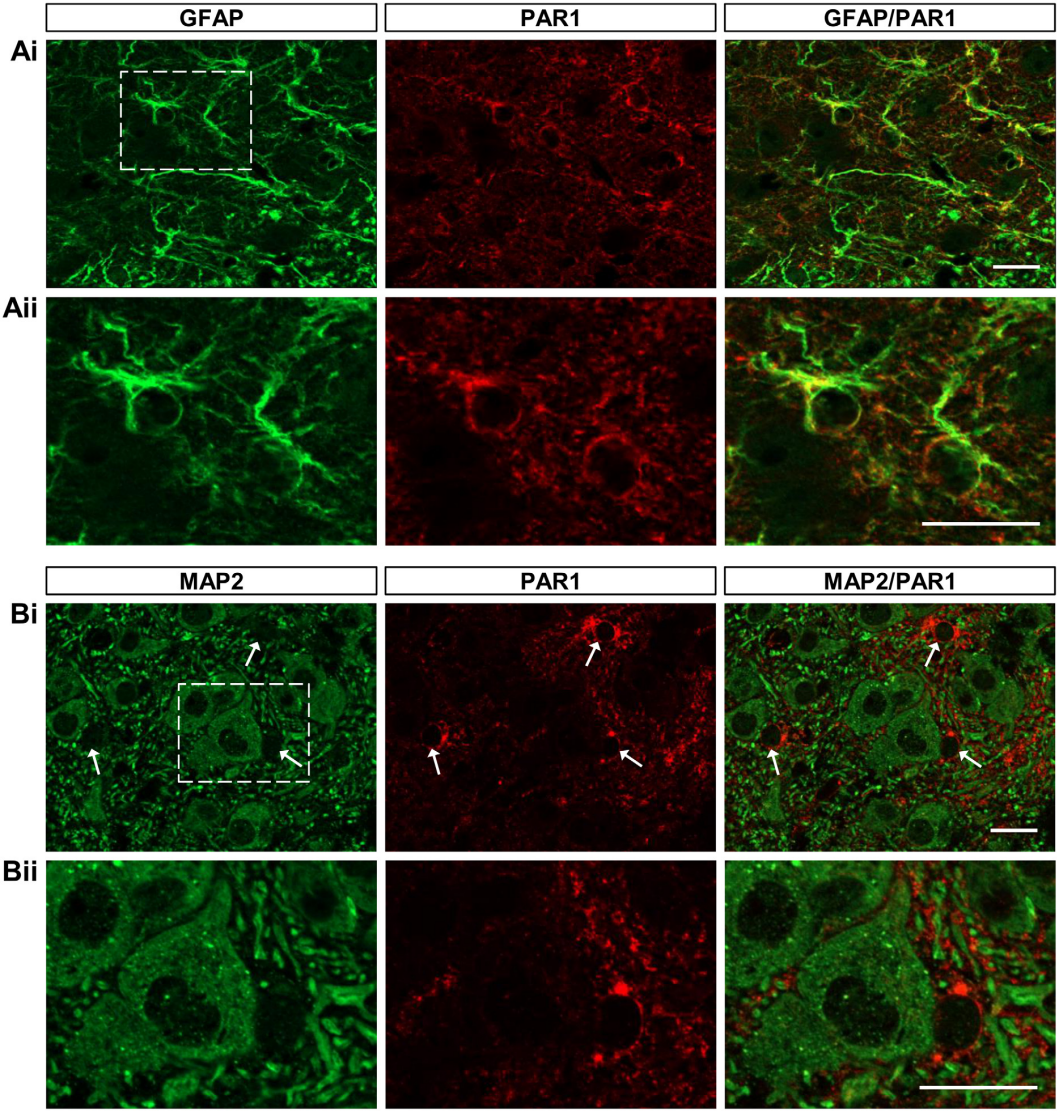
PAR1 immunoreactivity co-localises with GFAP but not with MAP2 in the lumbar ventral spinal cord. A: representative images showing 50 μm transverse sections taken from the upper lumbar spinal cord of a P6 C57BL/6 mouse. Sections were stained with antibodies raised against GFAP (green) and PAR1 (red). B: representative images showing 50 μm transverse sections taken from the upper lumbar spinal cord of a mouse. Sections were stained with antibodies raised against MAP2 (green) and PAR1 (red). Arrows in Bi indicate areas of PAR1 staining between MAP2+ cells. Scale bars: 20 μm.

Protease activated receptor-1 (PAR1), an endogenous G-protein coupled receptor (GPCR), is preferentially expressed by astrocytes in the brain and brainstem [43,44], and application of the peptide agonist TFLLR has been shown to elicit Ca^2+^ signalling selectively in cortical astrocytes [33,34,45]. To assess the suitability of PAR1 activation as a strategy for selectively stimulating glia during ongoing rhythmic activity of spinal motor networks *in vitro*, we first examined PAR1 expression in the spinal cord. In spinal cord slices taken from segments L_1_-L_3_ of P4-11 mice, PAR1 immunoreactivity co-localised with the astrocyte marker GFAP throughout the ventral horn (Rexed’s laminae VII-IX; Fig 1A; *n* = 4). By contrast, PAR1 immunoreactivity was not exhibited by cells positive for the pan-neuronal marker MAP2 (Fig 1B; *n* = 4), but was instead restricted to cells located between those labelled with MAP2 (Fig 1Bii, arrows). This pattern of expression, which was consistent across P4-11 tissue, resembles that previously reported in the brain and brainstem and supports the use of endogenous PAR1 for the specific stimulation of Ca^2+^-dependent processes in spinal cord glia [43,44].

**Fig 2:**
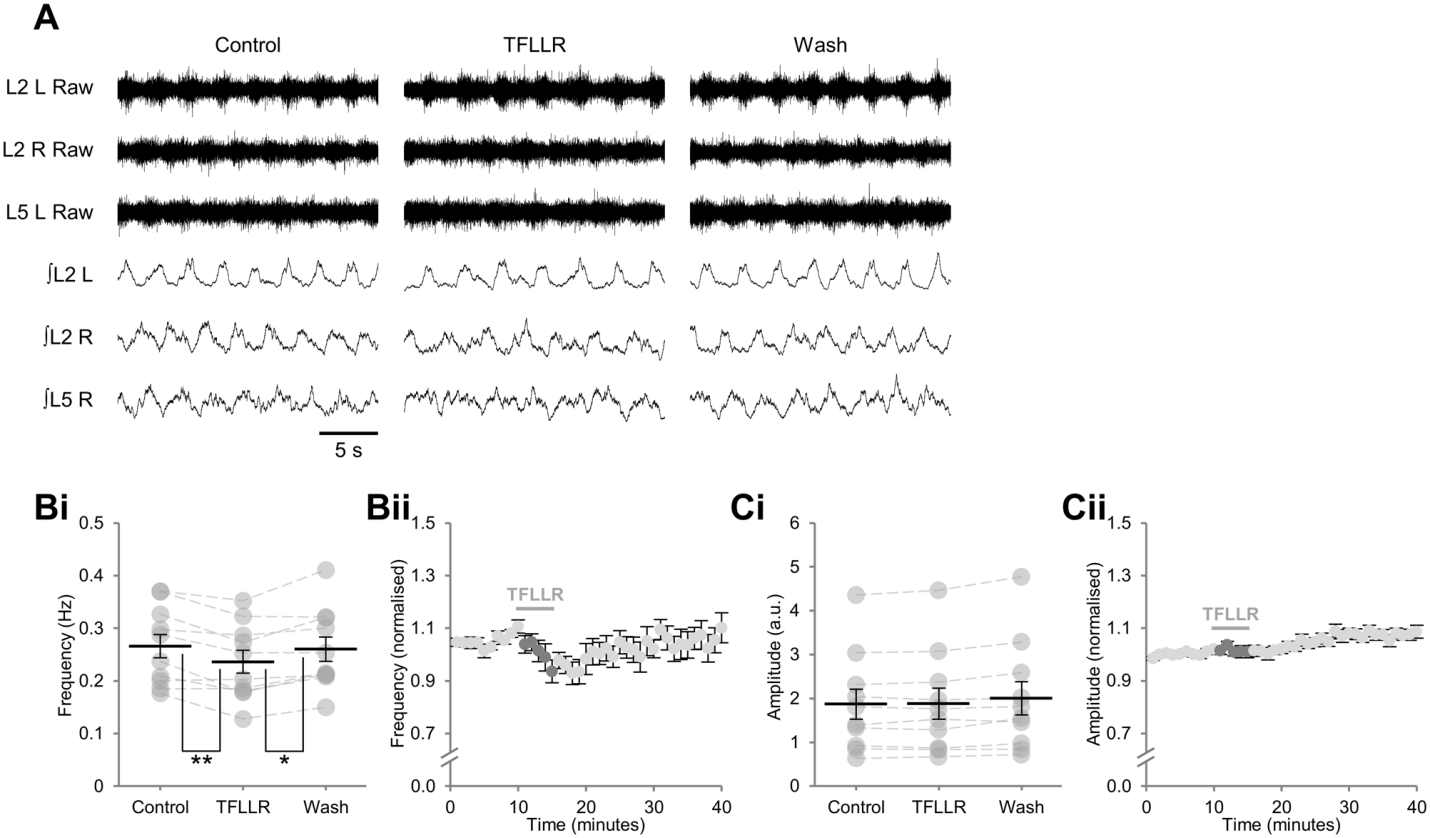
Stimulation of glia during ongoing locomotor-related activity results in a transient reduction in burst frequency. A: raw (top) and rectified/integrated (bottom) traces recorded from left (L) and right (R) L_2_ ventral roots and the right L_5_ ventral root showing the effect of the PAR1 agonist TFLLR (10 μM). Bi: locomotor-burst frequency over 3 min during a control period, immediately following TFLLR application, and following a 20-min washout period. Individual data points are shown in grey and means are represented by black lines. Bii: time course plot of normalised data aggregated into 1-min bins showing a reduction in burst frequency upon application of TFLLR. Ci: locomotor-burst amplitude over 3 min during a control period, immediately following TFLLR application, and following a 20-min washout period. Cii: time course plot of normalised data aggregated into 1-min bins showing no change in burst amplitude upon application of TFLLR. *n* = 10 preparations. Statistically significant differences in pairwise comparisons: *p < 0.05, **p < 0.01.

To determine the contribution of glial-neuronal signalling to the modulation of the mammalian spinal circuitry controlling locomotion, we made a brief (5 min) bath application of the PAR1-specific agonist TFLLR (10 μM) [33,34] to isolated mouse spinal cord preparations while recording ongoing pharmacologically induced (10 μM 5-HT, 50 μM DA, 5 μM NMDA) fictive locomotor-related activity from lumbar ventral roots (Fig 2A). TFLLR caused a transient reduction in the frequency of locomotor-related bursting, beginning within the first minute of application, and with a maximum effect of 11.5 ± 2.9% after 5 min (Fig 2A and 2B; *F*[2,18] = 9.1, *p* < 0.01, *d* = 0.43, *n* = 10). Burst duration was found to increase during TFLLR application (21.0 ± 6.7%; *F*[2,18] = 9.6, *p* < 0.01, *n* = 10) whereas duty cycle did not change significantly (*F*[2,18] = 0.8, *p* > 0.05, *n* = 10). In addition, TFLLR application had no effect on the peak-to-peak amplitude of bursts (Fig 2C; *F*[2,18] = 3.9, *p* > 0.05, *n* = 10). Alternation of bursts, both between contralateral roots and between ipsilateral roots L_2_ and L_5_, was maintained throughout the drug application and wash periods (Fig 2A).

**Fig 3:**
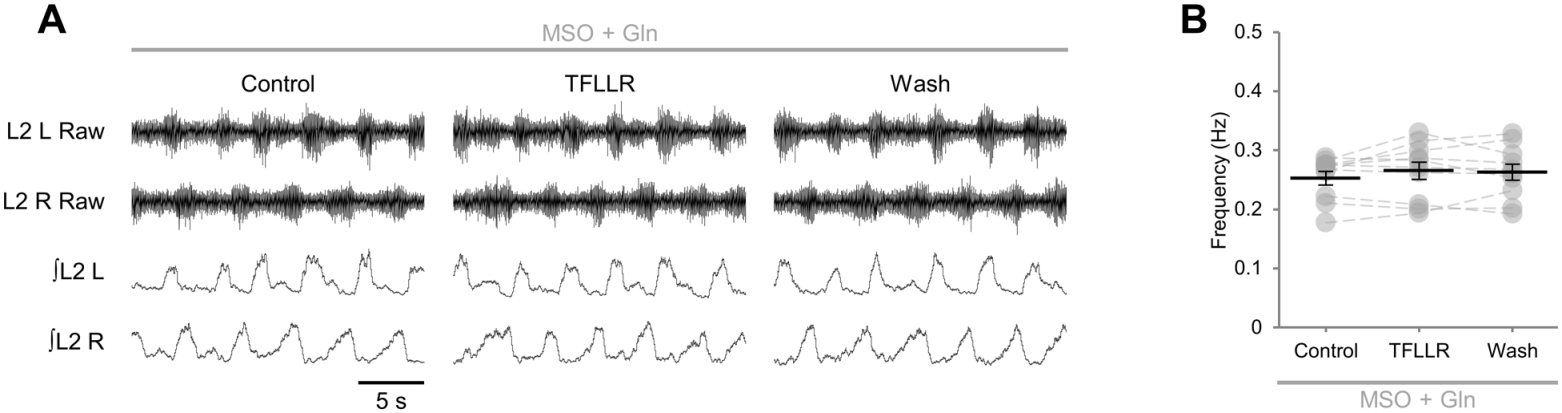
TFLLR has no effect on locomotor-related bursting following pharmacological ablation of glia. A: raw (top) and rectified/integrated (bottom) traces recorded from left (L) and right (R) L_2_ ventral roots showing the effect of the PAR1 agonist TFLLR (10 μM) following glial ablation with methionine sulfoximine (MSO; 100 μM), which was co-applied with glutamine (Gln; 1.5 mM). B: locomotor-burst frequency in the presence of MSO and Gln over 3 min during a control period, immediately following TFLLR application, and following a 20-min washout period. Individual data points are shown in grey and means are represented by black lines. *n* = 10.

To confirm that the reduced frequency of locomotor-related bursting detected upon TFLLR application was mediated by glia, and that TFLLR had no direct effects on neurons, we applied TFLLR to preparations in which glia had been ablated by exposure to gliotoxins [10,12,46]. Stable locomotor-like bursting resembling that observed in the absence of toxins persisted following application of MSO (100 μM) or FA (5 mM), both of which selectively disrupt glial metabolism, when the aCSF was supplemented with glutamine (1.5 mM) to sustain synthesis of glutamate and GABA [10,12,46]. Burst frequency was found to be unaffected by TFLLR applied 1 hr after either MSO (Fig 3A and 3B; *F*[2,18] = 1.0, *p* > 0.05, *n* = 10) or FA (5 mM; *F*[2,18] = 0.2, *p* > 0.05, *n* = 10), indicating that the effect of TFLLR on locomotor network activity is mediated by glia and that TFLLR does not directly affect neurons. Together these findings imply that, when stimulated via an endogenous GPCR, spinal cord glia are capable of releasing a factor or factors that reduce the frequency of rhythmic activity generated by locomotor circuits.

### Network modulation following PAR1 activation is mediated by adenosine derived from ATP

**Fig 4:**
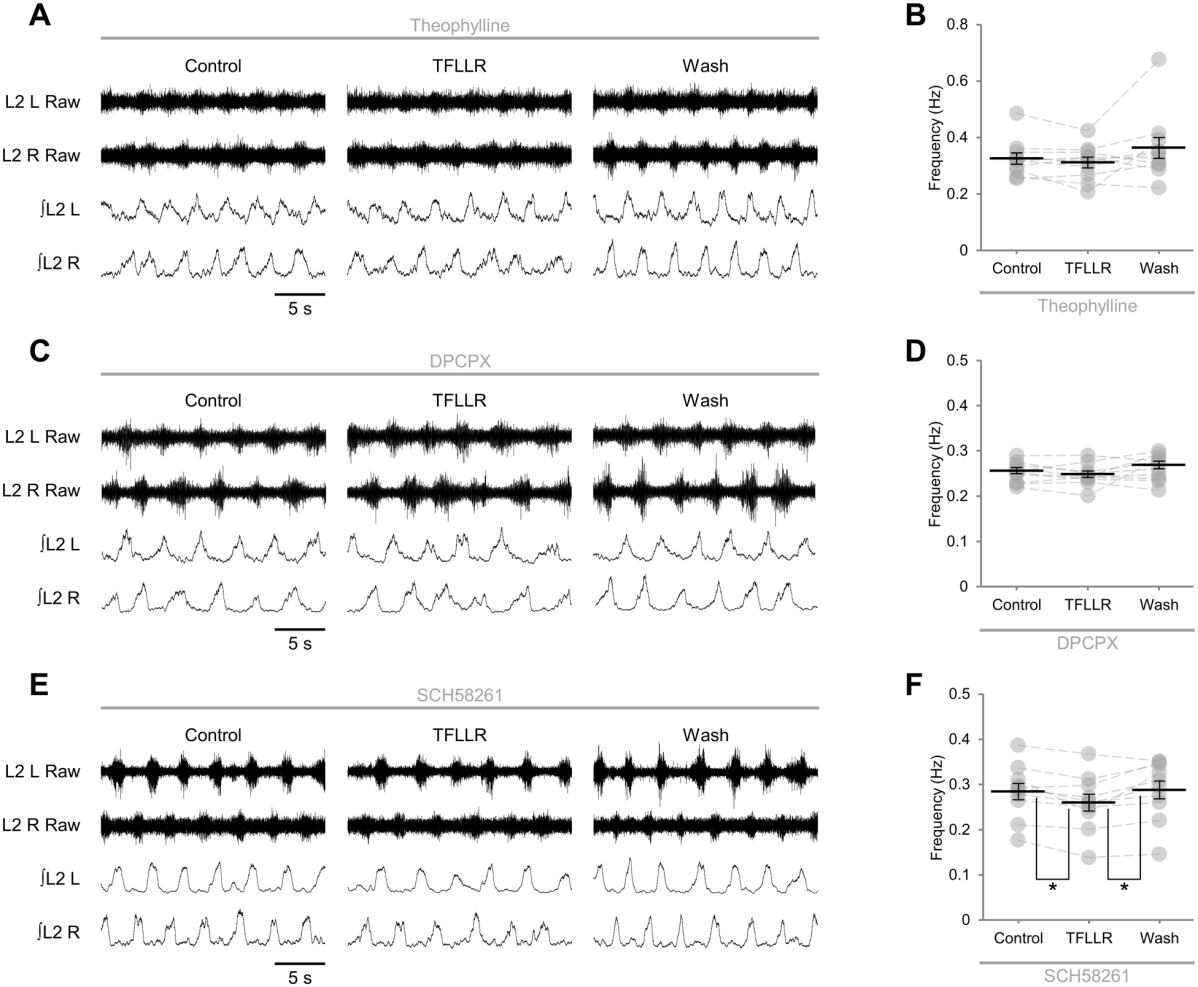
Glial stimulation results in release of adenosine and activation of neuronal A_1_ receptors. A: raw (top) and rectified/integrated (bottom) traces recorded from left (L) and right (R) L_2_ ventral roots showing the effect of the PAR1 agonist TFLLR (10 μM) applied in the presence of theophylline (20 μM). B: locomotor-burst frequency in the presence of the non-selective adenosine receptor antagonist theophylline over 3 min during a control period, immediately following TFLLR application, and following a 20-min washout period (*n* = 10). C: raw (top) and rectified/integrated (bottom) traces recorded from left (L) and right (R) L_2_ ventral roots showing the effect of the PAR1 agonist TFLLR (10 μM) applied in the presence of the A_1_-receptor antagonist 8-cyclopentyl-1,3-dipropylxanthine (DPCPX; 50 μM). D: locomotor-burst frequency in the presence of DPCPX over 3 min during a control period, immediately following TFLLR application, and following a 20-min washout period (*n* = 10). E: raw (top) and rectified/integrated (bottom) traces recorded from left (L) and right (R) L_2_ ventral roots showing the effect of the PAR1 agonist TFLLR (10 μM) applied in the presence of SCH58261 (25 μM). F: locomotor-burst frequency in the presence of the A_2A_-receptor antagonist SCH58261 over 3 min during a control period, immediately following TFLLR application, and following a 20-min washout period (*n* = 10). Individual data points are shown in grey and means are represented by black lines. Statistically significant differences in pairwise comparisons: *p < 0.05.

We next sought to determine the identity of the neuromodulatory factor or factors released by glia upon PAR1 activation. We have previously shown that spinal motor networks are modulated by endogenous adenosine that appears to derive from glia [12]. We therefore investigated whether adenosine mediated a component of the network response to PAR1 activation by applying TFLLR in the presence of the non-selective adenosine receptor antagonist theophylline (20 μM). In these preparations TFLLR had no effect on the frequency of locomotor-related bursting (Fig 4A and 4B; *F*[2,18] = 2.8, *p* > 0.05, *n* = 10), implying that adenosine is the dominant modulator released by glia following PAR1 activation. To investigate the adenosine receptor subtypes activated by glial cell-derived adenosine, we applied TFLLR in the presence of antagonists selective for either A_1_ or A_2A_ receptors, both of which are broadly expressed high-affinity adenosine receptor subtypes [14]. Like theophylline, the A_1_-subtype specific antagonist DPCPX (50 μM) efficiently abolished the modulation of burst frequency following PAR1 activation (Fig 4C and 4D; *F*[2,18] = 1.9, *p* > 0.05, *n* = 10). By contrast, in the presence of the A_2A_-subtype specific antagonist SCH58261 (25 μM), PAR1 activation caused a transient reduction in the frequency of locomotor-related bursting of a similar magnitude to that measured in the absence of receptor antagonists (9.1 ± 2.3%; Fig 4E and 4F; *F*[2,18] = 7.0, *p* < 0.01, *d* = 0.43, *n* = 10). In addition, PAR1 activation in the presence of SCH58261 did not affect burst amplitude (*F*[2,18] = 0.267, *p* > 0.05, *n* = 10).

**Fig 5:**
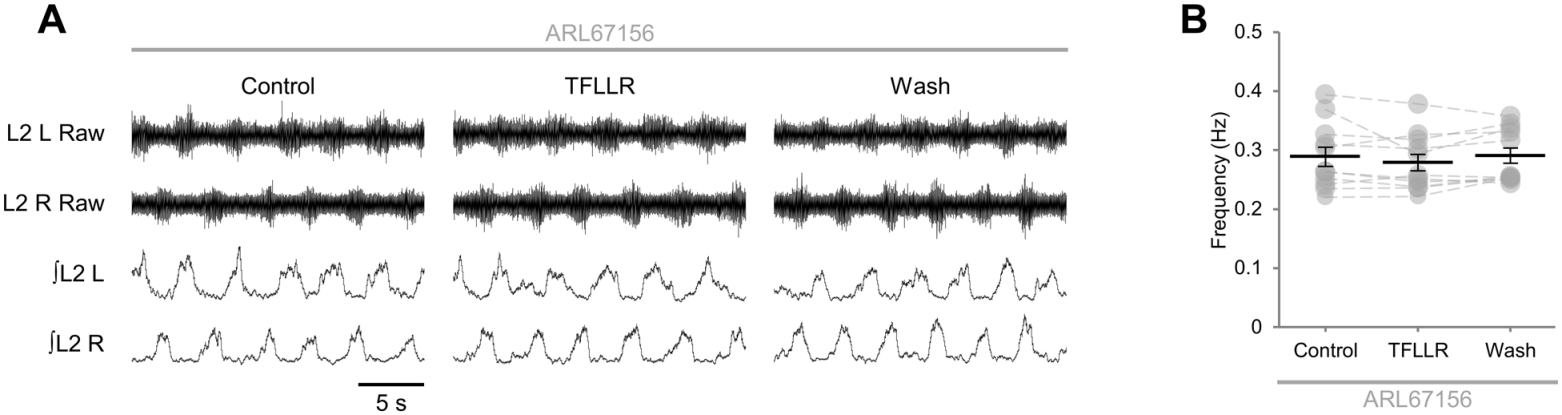
Modulation of locomotor network output upon glial stimulation requires extracellular degradation of ATP to adenosine. A: raw (top) and rectified/integrated (bottom) traces recorded from left (L) and right (R) L_2_ ventral roots showing the effect of the PAR1 agonist TFLLR (10 μM) applied in the presence of the ectonucleotidase inhibitor ARL67156 (50 μM). B: locomotor-burst frequency in the presence of ARL67156 (50 μM) over 3 min during a control period, immediately following TFLLR application, and following a 20-min washout period (*n* = 11).

Adenosine may be released from cells directly or else result from the ectonucleotidase-mediated hydrolysis of ATP following its release into the extracellular space [14–16]. To further investigate the release of adenosine by glia during network activity, we applied TFLLR in the presence of the ectonucleotidase inhibitor ARL67156 (50 μM). In preparations to which ARL657156 was pre-applied, PAR1 activation did not modulate the frequency of locomotor-related bursting (Fig 5A and 5B; *F*[2,20] = 1.7, *p* > 0.05, *n* = 11), implying that glia do not release adenosine directly, but instead release ATP, which is subsequently degraded to adenosine. Together these data indicate that glia associated with spinal motor control networks release ATP following PAR1 activation, and that the adenosine produced by hydrolysis of ATP in the extracellular space acts on neuronal A_1_ but not A_2A_ receptors to inhibit locomotor-related activity.

### Glial cell-derived adenosine does not modulate excitatory components of the locomotor circuitry

**Fig 6:**
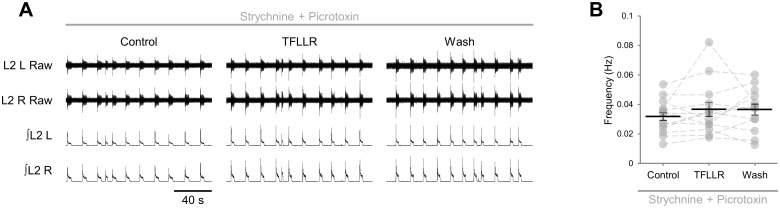
ATP-adenosine released following stimulation of glia modulates inhibitory components of locomotor networks. A: raw (top) and rectified/integrated (bottom) traces recorded from left (L) and right (R) L_2_ ventral roots showing the effect of the PAR1 agonist TFLLR (10 μM) applied to preparations in which inhibitory transmission was blocked by the GABA_A_-receptor antagonist pictrotoxin (10 μM) and the glycine-receptor antagonist strychnine (1 μM). B: locomotor-burst frequency in disinhibited preparations over 6 min during a control period, immediately following TFLLR application, and following a 20-min washout period (*n* = 13). Individual data points are shown in grey and means are represented by black lines.

Having determined that PAR1 activation results in the release of ATP-adenosine from glia, we sought to identify the cellular targets of glial cell-derived adenosine within spinal motor circuitry. To investigate the relative sensitivity of excitatory versus inhibitory components of spinal motor networks to the adenosine produced upon PAR1 activation, we applied TFLLR to disinhibited preparations in which inhibitory transmission was blocked by the glycine receptor antagonist strychnine (1 μM) and the GABA_A_ channel antagonist picrotoxin (60 μM) [12,32]. Blockade of inhibitory transmission results in slow (0.032 ± 0.003 Hz, *n* = 13), rhythmic, large-amplitude bursts that are synchronous across all ventral roots (Fig 6A). The frequency of these bursts was unchanged following a 10-min bath application of TFLLR (Fig 6A and 6B; *F*[2,24] = 1.2, *p* > 0.05, *n* = 13). Similarly, burst amplitude was unaffected following glial stimulation (*F*[2,24] = 2.294, *p* > 0.05, *n* = 13). These results suggest that the adenosine produced upon PAR1 activation primarily modulates the activity of inhibitory interneurons or microcircuits not active within disinhibited preparations.

### Glial cell-derived adenosine mediates feedback inhibition of locomotor-network activity

**Fig 7:**
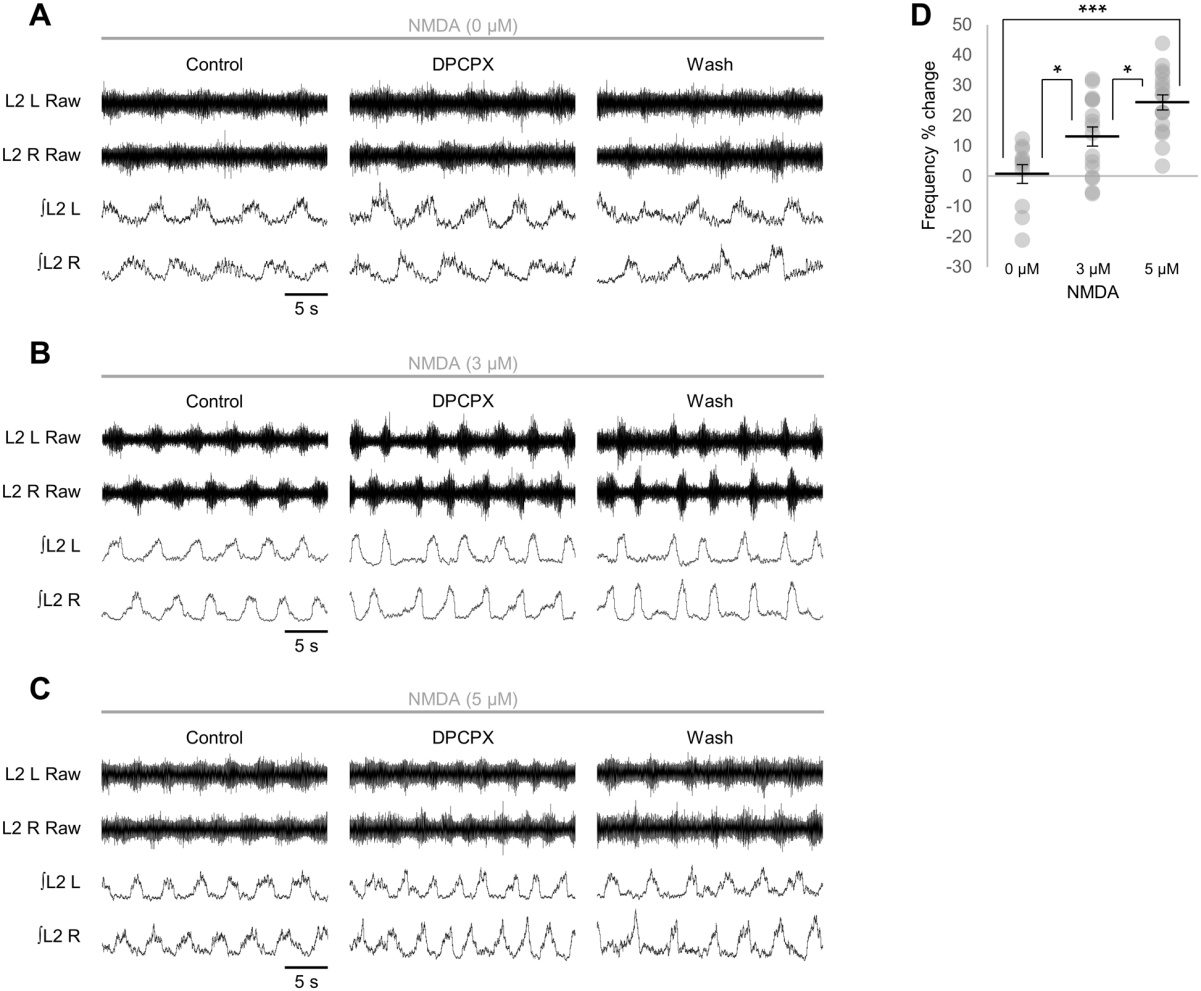
Adenosinergic modulation of locomotor networks scales with network activity. A-C: raw (top) and rectified/integrated (bottom) traces recorded from left (L) and right (R) L_2_ ventral roots showing the effect of the A_1_-receptor antagonist DPCPX (50 μM) in preparations in which fictive locomotion was evoked by 5-HT (10 μM) and DA (50 μM) alone (A) or with 3 μM NMDA (B) or 5 μM NMDA (C). D: percentage change in locomotor-burst frequency in response to DPCPX application in preparations in which fictive locomotion was evoked at different frequencies using 0 μM NMDA (*n* = 11), 3 μM NMDA (*n* = 16) and 5 μM NMDA (*n* = 17), calculated by comparing a 10-min control period with the last 10 min of a 30-min application of DPCPX. Individual data points are shown in grey and means are represented by black lines. Statistically significant differences in pairwise comparisons: *p < 0.05, ***p < 0.001.

Finally, we considered whether modulation of the murine locomotor CPG by endogenous glial cell-derived adenosine scales with network activity, as predicted by the tripartite synapse model of bidirectional signalling between neurons and glia [1,30]. Stable locomotor-related bursting was generated by bath application of 5-HT (10 μM), DA (50 μM) and three different concentrations of NMDA to generate a range of control frequencies (0 μM NMDA: 0.097 ± 0.017 Hz, *n* = 11; 3 μM NMDA: 0.131 ± 0.008 Hz, *n* = 16; 5 μM NMDA: 0.175 ± 0.001 Hz, *n* = 14) [47] and the effect of endogenous adenosine at each level of network activity was revealed by application of DPCPX (50 μM) to block A_1_ receptors, as previously described [12]. The change in frequency of locomotor-related bursting following DPCPX application increased with NMDA concentration (Fig 7A–7D; *F*[2,41] = 14.2, *p* < 0.001, *n* = 11–17): the frequency of locomotor-related bursting following DPCPX application was not significantly altered with 0 μM NMDA (3.1 ± 4.4%; Fig 7A and 7D; *F*[2,20] = 1.0, *p* > 0.05, *n* = 11) but increased by 13.1 ± 3.1% with 3 μM NMDA (Fig 7B and 7D; *F*[2,30] = 6.1, *p* < 0.05, *n* = 16) and by 24.4 ± 2.5% with 5 μM NMDA (Fig 7C and 7D; *F*[2,32] = 11.9, *p* < 0.01, *n* = 17). To confirm that the effect of endogenous adenosine was related to the baseline frequency of locomotor-related activity and was not instead mediated by a direct action of NMDA on glia, we also compared the effect of DPCPX on low-frequency activity generated by 10 μM 5-HT and 50 μM DA in standard aCSF containing 3 mM K^+^ with its effect on activity with a higher baseline frequency (0.24 ± 0.017 Hz, *n* = 6) induced by 5-HT and DA at the same concentrations in aCSF containing 5 mM K^+^. In high-K^+^ solution DPCPX raised burst frequency by 23.0 ± 6.6% (*F*[2,10] = 8.7, *p* < 0.01, *n* = 6), differing significantly from its effect in 3 mM K^+^ solution (Student’s *t*-test, *p* < 0.05, *n* = 6–11). Together these findings indicate that the influence of adenosine on locomotor-related bursting scales with the level of network activity, likely reflecting detection of neural activity by glia and a proportional release of ATP-adenosine.

## Discussion

We provide evidence that glia contribute to the operation of spinal motor networks by the secretion of neuromodulatory ATP-adenosine in an activity-dependent manner. This study extends our previous characterisation of the modulation of network output by purines and corroborates our proposal that glia are the source of neuromodulatory adenosine in the spinal motor circuitry [12]. Furthermore, our findings suggest that adenosine is the primary, if not sole, gliotransmitter responsible for the modulation of mouse spinal locomotor networks.

Glia have been shown to release a number of substances including glutamate, D-serine, ATP and GABA upon experimental stimulation in both the brain and spinal cord, with diverse effects on synaptic transmission [1,30]. Stimuli are proposed to reproduce the cytosolic Ca^2+^ elevations evoked in glia following the activation of endogenous receptors by transmitters released during neuronal activity, a phenomenon that has been observed throughout the CNS, both *in vitro* and *in vivo* [2,4]. We show that experimental stimulation of glia by activation of PAR1 during ongoing locomotor-like activity within spinal motor networks results in a reversible reduction in the frequency of locomotor-related bursting associated with an increase in burst duration, with no effect on cycle period or burst amplitude.

PAR1 is an endogenous G-protein coupled receptor associated with G_q_ proteins, and its activation has been shown to result in the release of Ca^2+^ from internal stores in cortical slice preparations, followed by Ca^2+^-dependent release of glutamate or ATP [33,34,45]. Similar glial cell-specific effects of PAR1 activation have been proposed in the ventral horn of the spinal cord [20]. We demonstrate preferential expression of PAR1 by GFAP+ cells in the spinal cord, consistent with findings from the brain and brainstem [44,43], and supporting its use to stimulate glia in intact spinal cord preparations. Although we do not detect clear PAR1 immunofluorescence in MAP2+ neurons, our data cannot exclude the possibility of low-level PAR1 expression by neurons. To confirm that the effects of PAR1 activation on network output were mediated by glia and that TFLLR had no off-target effects on neurons, we apply the PAR1-selective agonist TFLLR following pharmacological ablation of glia by FA and MSO. Toxins that disrupt normal glial metabolism have been used in several studies to elucidate the role of glia in neuromodulation and homeostasis [10,12,16,48–51]. Although both MSO, an inhibitor of glutamine synthetase [52] and FA, a precursor of the aconitase inhibitor fluorocitrate [49], are able to cross neuronal membranes [53] and have actions that may not result directly from the disruption of glial metabolism [48,54,55], neither prevents the rhythmic activity of motor networks when co-applied with glutamine to ensure continued synthesis of glutamate and GABA [10,12,46]. Furthermore, FA and MSO do not prevent the modulation of motor networks by exogenously applied neuromodulators [10,12]. We therefore propose that the modulation of locomotor-network output by TFLLR reflects selective activation of PAR1 expressed by spinal glia rather than any off-target effects on neurons, consistent with previous reports that TFLLR does not act on neurons in the brain [33,34].

The effects of PAR1 activation on network output closely resemble those of exogenously applied adenosine, which likewise depresses the frequency of locomotor-related bursting without modulating burst amplitude or left-right alternation [12]. In support of A_1_ adenosine receptor-mediated modulation of the locomotor CPG following PAR1 activation, the modulation of network output is efficiently prevented by either the general adenosine-receptor antagonist theophylline or the A_1_ antagonist DPCPX, but not by the A_2A_ antagonist SCH58261. Similarly, inhibition of A_1_ receptors, but not A_2A_ receptors is sufficient to abolish the modulation of fictive locomotion by endogenous adenosine [12], and DPCPX abolishes the PAR1-evoked modulation of excitatory currents recorded from ventral horn interneurons [20]. Of the four known adenosine receptor subtypes, A_1_ and A_2A_ receptors have the highest affinity for adenosine, and in many regions A_1_-mediated inhibition balances A_2A_-mediated facilitation [14]. Although both receptors are expressed throughout the spinal cord [56–58], A_2A_ receptors do not appear to modulate the activity of the locomotor CPG (Fig 4E and 4F) [12].

Given that glia have been shown in numerous systems to be competent to release a range of modulators [1,30], and that a wide range of neuromodulators has been shown to regulate spinal motor networks [9], it may be surprising that PAR1 stimulation of glia results in the modulation of locomotor network activity by adenosine alone. Although glia have been proposed to exercise fine control over synaptic transmission [59–61], our findings suggest that the diversity of neuronal signalling mechanisms in the spinal cord is not reflected by a similar diversity of gliotransmitters. Instead, adenosine derived from glia appears to provide broad negative feedback control of locomotor networks. It should, however, be noted that the choice of technique employed to stimulate glia has been found to influence gliotransmitter release [45,62] and for this reason we cannot exclude the possibility that spinal glia release other gliotransmitters in response to different stimuli.

Under non-pathological conditions, extracellular adenosine is largely derived from the hydrolysis of ATP by ectonucleotidases [63]; however, it may also be released directly via exocytosis or equilibrative nucleoside transporters [15,16]. Several studies have detected Ca^2+^-dependent release of ATP by glia, with subsequent conversion of ATP to adenosine and activation of either A_1_ or A_2A_ receptors [17–20]. We show that the reduction in the frequency of locomotor-like bursting detected following PAR1 activation is abolished in the presence of the ectonucleotidase inhibitor ARL67156, implying that ATP and not adenosine is released by glia following stimulation. In other networks including the mammalian respiratory CPG [25] and tadpole locomotor CPG [21], modulation of neuronal activity by adenosine opposes modulation by ATP. By contrast, the present study indicates that spinal motor networks in the mouse are modulated by adenosine derived from the extracellular hydrolysis of ATP, but not by ATP directly, a finding supported by our previous investigation of purinergic modulation of the murine locomotor CPG [12].

Stimulation of glia does not influence the frequency of synchronous, disinhibited bursting generated and coordinated exclusively by excitatory components of the motor circuitry, consistent with our previous finding that exogenously applied adenosine has no effect on disinhibited bursting [12]. We have previously demonstrated that disinhibited bursting remains sensitive to subtle neuromodulatory influences [64], suggesting that PAR1 activation would result in a measurable effect on disinhibited bursting if the excitatory neurons underlying this activity were sensitive to modulation by adenosine. Although it remains uncertain whether common circuit elements underlie both disinhibited and left-right alternating rhythms, the data presented here imply that adenosine acts on inhibitory interneurons to modulate the frequency of locomotor-related bursting, a conclusion supported by our recent finding that adenosine modulates inhibitory transmission onto ventral horn interneurons [65]. A model in which adenosine acts on inhibitory interneurons to modulate locomotor-related activity may appear to contradict the recent finding by Carlsen and Perrier (2014) [20] that glial cell-derived adenosine modulates excitatory transmission onto ventral horn interneurons. However, an indirect mechanism whereby adenosine modulated excitatory drive to inhibitory interneurons could also explain the loss of modulation in disinhibited preparations. Because adenosine does not affect the contralateral alternation of locomotor-related bursts, we have previously proposed that adenosine may modulate the activity of ipsilaterally projecting inhibitory interneurons, of which V1 local circuit inhibitory neurons are an example [12,66]. The ablation or inactivation of V1 interneurons results in a reduction in the frequency of locomotor-like activity [67], making them a candidate for future studies aiming to decipher the cellular targets of modulatory adenosine. Modulation of V1 or a similar population of ipsilaterally projecting inhibitory interneurons could reflect either a direct action of adenosine on those neurons or an indirect action on the excitatory inputs they receive. Future experiments should seek to identify directly the population of neurons on which glial cell-derived adenosine acts to inhibit locomotor-related activity and the mechanism or mechanisms by which its action on individual neurons translates to regulation of the frequency of rhythmic activity at the network level.

Finally, we provide evidence that the influence of endogenous adenosine on network output increases with the frequency of network activity, likely reflecting enhanced release of ATP-adenosine from glia and implying a mechanism for the detection of neuronal activity by glia. In other preparations glial Ca^2+^ signalling is triggered by the spillover of neurotransmitters from synapses [30]. A similar mechanism operating in the spinal cord may therefore involve the spillover of glutamate, glycine or GABA, all of which are endogenous neurotransmitters to which spinal glia have been shown to respond [68,69].

Activity-dependent increases in extracellular adenosine have previously been detected in several preparations [70]. Of particular relevance to this study is the *Xenopus* tadpole spinal cord, in which ATP is released during swimming episodes and enhances the excitability of motor networks [21]. The hydrolysis of ATP by ectonucleotidases as swimming proceeds raises the concentration of extracellular adenosine, which opposes ATP to reduce network excitability and terminate swimming. This entails the activation of A_1_ receptors and a reduction in voltage-gated Ca^2+^ currents without a change in the strength of synaptic transmission. By contrast, in murine spinal locomotor networks, ATP does not appear to act as a neuromodulator, whereas activation of A_1_ receptors depresses synaptic transmission by presynaptic inhibition of neurotransmitter release [12,20] and hyperpolarises the resting membrane potential of ventral horn interneurons [65]. Since ATP is consumed by neurons during activity, raising cytoplasmic adenosine levels, an efficient coupling mechanism would entail direct release of adenosine via neuronal equilibrative nucleoside transporters, with autocrine inhibition of activity via A_1_ receptors [13,14]. This mechanism does not appear to operate in mouse spinal motor networks or other systems in which the source of adenosine is ATP released into the extracellular space from either neurons or glia [12,70]. Whether adenosine production is direct or indirect, the functional consequences of the negative feedback it provides may include the stabilisation of network activity and the prevention of excitotoxicity and metabolic exhaustion.

Despite considerable evidence from *in vitro* brain preparations that glia sense activity in neighbouring neurons and, in turn, modulate that activity in a Ca^2+^-dependent manner, there is a paucity of evidence that gliotransmission is important for the operation of brain networks and the behaviours they direct [4–6]. Rhythmically active CPGs in the brainstem and spinal cord provide a tractable model for studying the contribution of gliotransmission to network output and thus behaviour. In brainstem preparations it has been shown that glia mediate purinergic modulation of rhythmically active inspiratory networks, most likely owing to the Ca^2+^-dependent release of glutamate [10], and that acidification of the extracellular medium triggers the release of ATP from glia in a Ca^2+^-dependent manner to stimulate activity in the phrenic nerve [11]. It has also recently been demonstrated that glia release the Ca^2+^-binding protein S100β in a Ca^2+^-dependent manner to confer rhythmic bursting properties on associated neurons in the brainstem circuity for mastication [71]. In the present study we provide evidence that activity-dependent release of ATP-adenosine from glia in the mammalian spinal cord provides negative feedback onto the circuitry that controls locomotion. This feedback may stabilise activity, delay metabolic rundown and/or have a neuroprotective role. Our findings inform understanding of the regulation of locomotor networks and provide an insight into the role of glia in shaping activity at the network level.
